# Type I PRMT Inhibition Protects Against C9ORF72 Arginine-Rich Dipeptide Repeat Toxicity

**DOI:** 10.3389/fphar.2020.569661

**Published:** 2020-09-08

**Authors:** Alan S. Premasiri, Anna L. Gill, Fernando G. Vieira

**Affiliations:** ALS Therapy Development Institute, Cambridge, MA, United States

**Keywords:** ALS (Amyotrophic lateral sclerosis), FTD (Fronto-Temporal Dementia), C9ORF72 DPRs, arginine methylation, protein arginine methyl transferase, glycine-arginine, proline-arginine

## Abstract

Repeat expansion mutations in the C9ORF72 gene are the most common genetic cause of amyotrophic lateral sclerosis (ALS) and frontotemporal dementia (FTD). Repeat-associated non-AUG translation of this expansion produces dipeptide repeat proteins (DRPs). The arginine containing DRPs, polyGR and polyPR, are consistently reported to be the most toxic. Here we demonstrated that small molecule inhibition of type I protein arginine methyltransferases (PRMT) protects against polyGR and polyPR toxicity. Furthermore, our findings suggest that asymmetric dimethylation of polyGR and polyPR by Type I PRMTs plays important roles in their cytotoxicity.

## Introduction

A mutation in the *C9orf72* gene is the most common known cause of amyotrophic lateral sclerosis (ALS) and frontotemporal dementia (FTD) (DeJesus-Hernandez et al., 2011; Renton et al., 2011). The mutation consists of an abnormal expansion of a repeated hexanucleotide sequence (GGGGCC) in the first intron of the *C9orf72* gene (DeJesus-Hernandez et al., 2011; Renton et al., 2011). In ALS and FTD, the expanded nucleotide tract is translated through an unconventional mechanism known as repeat-associated non-AUG (RAN) translation (Ash et al., 2013; Mori et al., 2013). Depending on what reading frame RAN translation takes place in, along either the sense or antisense RNA strand, it leads to the generation of five different dipeptide repeat proteins (DRPs) of variable lengths: poly-Glycine-Arginine (polyGR), poly-Proline-Arginine (polyPR), poly-Proline-Alanine (polyPA), poly-Glycine-Alanine (polyGA), and poly-Glycine-Proline (polyGP) (Ash et al., 2013; Mori et al., 2013).

The arginine-containing DRPs in particular have been demonstrated to have detrimental effects in several model systems and to interact with several different pathways (Kwon et al., 2014; Wen et al., 2014; Kramer et al., 2018). For example, when administered exogenously to U2OS cells, synthetic GR_20_ and PR_20_ are shown to bind to nucleoli, disrupt RNA splicing and processing, and decrease cell viability (Mori et al., 2013). Our lab has previously demonstrated that exogenous application of synthetic GR_15_ and PR_15_ to mouse spinal cord neuroblastoma hybrid cells (NSC-34) induces cellular toxicity, as measured by various cell health and function assays and that this toxic effect becomes more severe as the cells are further differentiated toward neurons, with primary neurons exhibiting the greatest toxicity (Gill et al., 2019). In addition, a series of studies involving the expression of the repeat expansion in *Drosophila* have demonstrated polyGR and polyPR related toxicity (Mizielinska et al., 2014; Freibaum et al., 2015; Lee et al., 2016), with one study revealing the disruption of stress granule assembly due to the presence of polyGR and polyPR (Lee et al., 2016). Other pathways that have been implicated in arginine-containing DRP toxicity include those involved in nucleocytoplasmic transport (Freibaum et al., 2015) and RNA-binding (Lee et al., 2016), though the complete nature of the pathogenesis of polyGR and polyPR remains unclear. Of particular interest, recent studies in ALS suggest a role for arginine methylation in disease progression and in polyGR-related toxicity (Ikenaka et al., 2019; Gittings et al., 2020).

Protein arginine methyltransferases (PRMTs) are a family of enzymes that post-translationally modify proteins by methylating nitrogen atoms of arginine residues. These modifications influence many cellular processes including transcription, RNA processing, signal transduction cascades, DNA damage response, and liquid-liquid phase separation (Guccione and Richard, 2019). Specifically, glycine- and arginine-rich (GAR) motifs, typical in histones and RNA binding proteins, are common targets for PRMT mediated modifications that are reported to influence protein localization and gene expression (Thandapani et al., 2013). In the present study we examined whether the cytotoxic effects of exogenously applied polyGR and polyPR would be affected by pharmacological inhibition of PRMT activity.

PRMTs are responsible for the monomethylation (MMe), asymmetric dimethylation (ADMe), and symmetric dimethylation (SDMe) of arginine residues, primarily within a GAR motif (Najbauer et al., 1993; Cheng et al., 2007) and are classified as type I, type II, or type III depending on the type of methylation they catalyze. Type I PRMTs catalyze ADMe with MMe as an intermediate, and include PRMT1, 2, 3, 4, 6 and 8. Type II PRMTs catalyze SDMe with MMe as an intermediate, and include PRMT5 and 9. Type III PRMTs perform MMe only and include PRMT7 (Blanc and Richard, 2017).

## Materials and Methods

### NSC-34 Cell Culture

NSC-34 cells (Cedarlane Laboratories, Burlington, ON, CA) were cultured in a complete medium consisting of high glucose Dulbecco’s modified eagle medium (DMEM) (Millipore-Sigma, Burlington, MA, USA) supplemented with 10% US-origin fetal bovine serum (Thermo Fisher Scientific, Cambridge, MA, USA), 1% 200 mM L-glutamine solution (Thermo Fisher Scientific, Cambridge, MA, USA), and 1% 10,000 U/mL penicillin-streptomycin solution (Thermo Fisher Scientific, Cambridge, MA, USA). Prior to preparation of NSC-34 complete medium, L-glutamine and penicillin-streptomycin solutions were aliquoted and stored at -20°C, and DMEM/high glucose was stored at 4°C. At each passage, cells were washed once with Dulbecco’s phosphate-buffered saline (DPBS) with calcium and magnesium (Thermo Fisher Scientific, Cambridge, MA, USA) and treated with 0.25% Trypsin-EDTA solution (Thermo Fisher Scientific, Cambridge, MA, USA) for 5 min at 37°C and 5% CO_2_ for dissociation. Prepared complete medium, DPBS, and Trypsin were always heated in a 37°C water bath before use and stored at 4°C between uses.

### Preparation of Exogenous Dipeptide Repeat Protein Solutions

Synthesized proteins GR_15_ (94.53% purity) and PR_15_ (93.17% purity) (GenicBio Limited, Kowloon, Hong Kong, CN) and ADMe-GR_15_ (95% purity) and ADMe-PR_15_ (98% purity) (Anaspec, Fremont, California, USA) were purchased as lyophilized powders and stored at -20°C in a desiccator prior to reconstitution. Proteins were reconstituted in sterile DMSO (Millipore-Sigma, Burlington, MA, USA) to stock concentrations of 10 mM and stored at 4°C.

### Preparation of PRMT Inhibitor Solutions

Small-molecule PRMT inhibitors MS023 and GSK591 (Tocris Bioscience, Briston, UK), MS049 and EPZ020411 (Cayman Chemical, Ann Arbor, MI, USA), GSK3368715 (Medchem Express, Monmouth Junction, NJ, USA), and negative control MS094 (Millipore-Sigma, Burlington, MA, USA) were purchased and stored at -20°C prior to and following reconstitution. After reconstitution using the solvents specified in **Table 1**, stocks were aliquoted to 15–20 µL and immediately stored at -20°C. UltraPure™ DNase/RNase-Free Distilled Water (Thermo Fisher Scientific, Cambridge, MA, USA.

**Table 1:** Small molecule PRMT inhibitors, controls, and solvents applied in these studies.

Drug name(s)	Vendor catalog #	Type of PRMT inhibitor	Solvent used to reconstitute	Stock concentration	Purity
GSK591GSK3203591EPZ015866	5777/10	Type II (symmetric)	DMSO	10 mM	≥97%
MS023	5713	Type I(asymmetric)	Water	10 mM	≥98%
MS094	SML2548	Inactive	DMSO	10 mM	99.5%
MS049	18348	Type I (asymmetric)	Water	10 mM	≥98%
EPZ020411	19160	Type I (asymmetric)	DMSO	10 mM	≥98%
GSK715GSK3368715EPZ019997	HY-128717A	Type I (asymmetric)	DMSO	10 mM	99.49%

### Small Molecule PRMT Inhibitor Details In Cell Western Assay

NSC-34 cells were seeded in clear, flat-bottom, full volume, 96-well tissue culture–treated plates (Thermo Fisher Scientific, Cambridge, MA, USA). After plating, cells were incubated for 24 h at 37°C and 5% CO_2_ prior to application of PRMT inhibitor or small molecule. Desired concentrations of compound were achieved by diluting aliquots of each stock into warmed culture medium. Cells were tested at least in quadruplicate. Once dosed, plates were incubated for 24h at 37°C and 5% CO_2_. After incubation, media was manually removed and fixed with 3.7% paraformaldehyde (Electron Microscopy Sciences, Hatfield PA, USA) in 1X PBS for 20 min at room temperature. Once fixed, fixing solution was removed manually and cells were permeabilized with 0.1% Triton X-100 + 1X PBS (Sigma-Aldrich, St. Louis, MO, USA) washes. Wells were blocked using Intercept Blocking Buffer (LI-COR, Lincoln, NE, USA) for 90 min at room temperature with shaking. Blocking buffer was manually removed and replaced with either anti-Asymmetric di-methyl arginine antibody (1:500, Cell Signaling Technology, 13522) or anti-symmetric di-methyl arginine antibody (1:800, Cell Signaling Technology, 13222) in intercept blocking buffer and kept at 4°C overnight with no shaking. After overnight incubation, antibody solution was manually removed and washed with 0.1% Tween 20 + 1X PBS. An IRDye 800CW goat anti-rabbit (1:1000, LI-COR) and CellTag700 (1:500, LI-COR) fluorescent antibody solution in intercept blocking buffer were added to the plate and incubated for 60 min at room temperature with shaking. Antibody solution was manually removed and washed with 0.1% Tween 20 + 1X PBS solution. Plate was read on the LI-COR Odyssey 9120 Infrared Imaging System. Data were expressed as a ratio of 800 channel signal to 700 channel signal (test condition to total protein).

### Plating NSC-34 and Dosing With DRPs and PRMT Inhibitors

NSC-34 cells were in clear, flat-bottom, full volume, 96-well tissue culture-treated plates (Thermo Fisher Scientific, Cambridge, MA, USA). One row on the top and bottom of the plate and two columns on either side of the plate were left without cells and contained culture medium only to minimize experimental well volume evaporation. After plating, cells were incubated for 24h at 37°C and 5% CO_2_ prior to DRP and/or PRMT inhibitor addition. At time of DRP/PRMT inhibitor addition, desired doses of DRP for challenge and inhibitor for treatment were achieved by diluting aliquots of each stock in warm culture medium. During experiments where both PRMT inhibitors and DRPs were used, PRMT inhibitors were always applied to wells first, followed by DRP application. Vehicle controls were included as wells treated with equivalent DMSO concentrations to those that had been DRP-treated, drug treated, or both. Inhibitor toxicity controls were included as wells treated with the desired doses of drug for the experiment, but no DRP. DRP toxicity controls were included as wells only treated with the doses of DRP used for challenge. Once dosed, plates were incubated for 24 h at 37°C and 5% CO_2_ prior to running the WST-1 or LDH assay endpoints. Additional controls needed for each endpoint are specified in the “WST-1 Assay” and “LDH Assay” sections of these methods.

### WST-1 Assay

Cells were plated and prepared using the steps described in the “Plating NSC-34 and Dosing with DRPs and PRMT Inhibitors” section of these methods. Other controls for this experiment included wells containing only cells in culture medium, and culture medium only. At time of testing, culture medium was removed from wells and replaced with a warmed, sterile-filtered solution consisting of DPBS with calcium and magnesium and 4.5 g/L of D-glucose (Millipore-Sigma, Burlington, MA, USA). To wells containing 200 µL of DPBS-glucose solution, 20 µL/well of WST-1 reagent (Millipore-Sigma, Burlington, MA) was applied, and plates were then incubated at 37°C and 5% CO_2_ for 1 h before plates were read at 450 nm on a SpectraMax M3 Microplate Reader (Molecular Devices, San Jose, CA, USA). As indicated in figures, WST-1 data was calculated relative to either GR/PR challenge or no challenge conditions using the following formulas. If DMSO was used as a solvent for compounds in test conditions, then same concentrations of DMSO were added to untreated controls.

$$\%MetabolicActivity=\frac{\left(A450TestCondition-A450DRPonlychallenge\right)}{\left(A450UntreatedControl-A450DRPonlychallenge\right)}*100\%$$

$$\%MetabolicActivity=\frac{\left(A450TestCondition\right)}{\left(A450UntreatedControl\right)}*100\%$$

### LDH Assay

Cells were plated and prepared using the steps described in the “Plating NSC-34 and Dosing with DRPs and PRMT Inhibitors” section of these methods. Other controls for this experiment included several sets of wells with only cells in culture medium (one triplicate designated for “untreated,” one triplicate designated for “lysed” positive control) and wells with culture medium only. An additional control added only to the transfer plate at time of testing was 5 µL of LDH only. Testing was performed using colorimetric LDH-Cytotoxicity Assay Kit II (Abcam, Cambridge, MA, USA) per manufacturer’s instructions. Final read at 450 nm was performed on a SpectraMax M3 Microplate Reader (Molecular Devices, San Jose, CA, USA). Data analysis included calculation of % LDH release using the following equation:

$$\%LDHRelease=\frac{\left(A450TestCondition-A450UntreatedControl\right)}{\left(A450LysedControl-A450UntreatedControl\right)}*100\%$$

### In Vitro Methylation (IVM) Assay

Individual assays were conducted in 0.5 mL, flat cap PCR tubes (Thermo Fisher Scientific, Cambridge, MA, USA). Systems contained recombinant PRMT1 (Active Motif, Carlsbad, CA, USA), S-(5-adenosyl)-L-methionine iodide (SAM, Sigma-Aldrich), either Histone H4 (Active Motif) or GR_15_ (Genic Bio), 10X PBS (Thermo Fisher Scientific), and nuclease-free water (Thermo Fisher Scientific). All reagents were added to achieve a final volume of 30 µl. Up to 0.8 µg of PRMT1, 25 µM of SAM, 3 µM of Histone H4, and 6.7 µM of GR_15_ were added to the system. Three microliters of 10X PBS was added to achieve 1X PBS. Once desired ratios of reagents were added to each tube, systems were lightly mixed and incubated for 2 h at 37°C and 5% CO_2_. After the incubation, reactions were stopped using 10 µl of 4x LDS Sample Buffer (Thermo Fisher Scientific).

### IVM Immunoblotting

After conducting the IVM assay, samples were boiled at 95°C for 5 min. Samples were separated on 4–12% Bis-Tris gels (Thermo Fisher Scientific) and blotted onto a nitrocellulose membrane. Membranes were blocked in a Superblock (Thermo Fisher Scientific) and Tween 20 solution and then incubated with rat anti-GR (1:1000, Millipore-Sigma, MABN778) and either rabbit anti-H4R3me2a (1:500, Active Motif, 39006) or rabbit anti-asymmetric di-methyl arginine antibody (1:500, Cell Signaling Technology, 13522). This was followed by incubation with fluorescently labeled IRDye antibodies (1:10,000 anti-rat 700 and 1:10,000 anti-rabbit 800, LI-COR 926-68076 and 925-32211) and read on the LI-COR Odyssey 9120 Infrared Imaging System. The protein standard used was the SeeBlue Plus2 Pre-stained protein Standard (Thermo Fisher Scientific LC5925).

### Statistics

**Figure 1:**
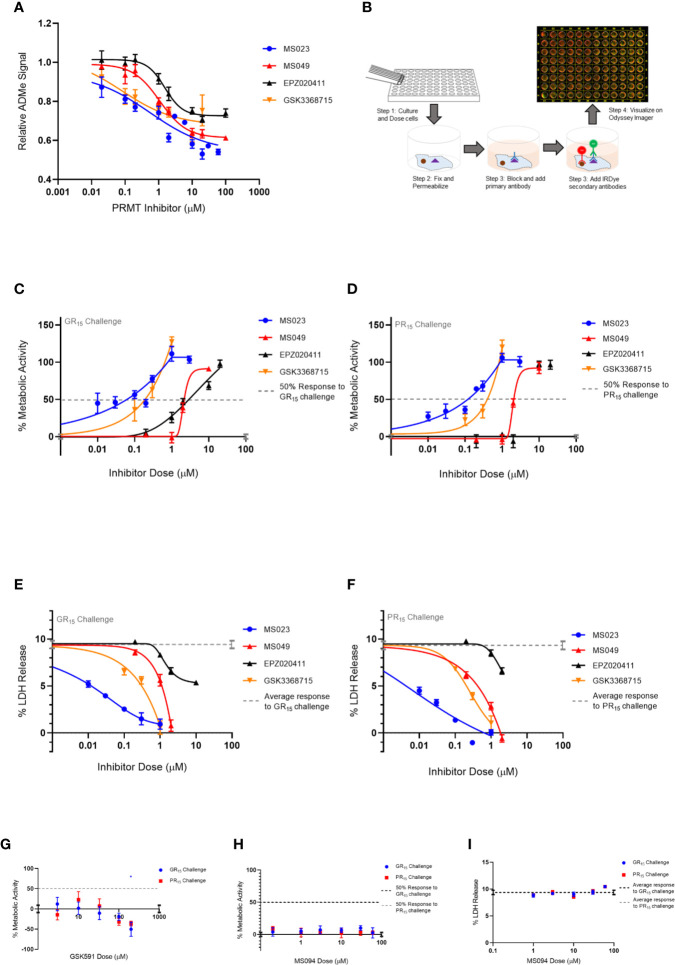
Type I PRMT inhibitors abrogate toxicity associated with GR_15_ and PR_15_ challenge in NSC-34 cells. **(A)** Relative signal of total ADMe in NSC-34 cells after having been incubated with Type I PRMT inhibitors for 24 hours. Quantification of the signal was done by ICW assay using an antibody against total ADMe and normalized using an antibody against total protein (MS023: df = 132, R^2^ = 0.7441; MS049: df = 82, R^2^ = 0.8218; EPZ020411: df = 83, R^2^ = 0.7087; GSK3368715: df = 50, R^2^ = 50). **(B)** Schematic of an ICW assay workflow and example visualization. We used a primary antibody against total ADMe, and a fluorescent green IRDye secondary antibody. A red, CellTag700 antibody was used to fluorescently label total protein. **(C, D)** Percent metabolic activity after challenging with 3 µM of GR_15_ or PR_15_ and dosing with a Type I PRMT inhibitor (GR_15_ challenge: MS023: df = 67, R^2^ = 0.8054; MS049: df = 10, R^2^ = 0.9630; EPZ020411: df = 13, R^2^ = 0.9237; GSK3368715: df = 19, R^2^ = 0.8077; PR_15_ challenge: MS023: df = 40, R^2^ = 0.9034; MS049: df = 10, R^2^ = 0.9571; EPZ020411: df = 13, R^2^ = 0.9625; GSK3368715: df = 7, R^2^ = 0.9330). **(E, F)** Percent LDH release after challenging with 3 µM of GR_15_ or PR_15_ and dosing with a Type I PRMT inhibitor (GR_15_ challenge: MS023: df = 22, R^2^ = 0.9056; MS049: df = 10, R^2^ = 0.9414; EPZ020411: df = 13, R^2^ = 0.8807; GSK3368715: df = 10, R^2^ = 0.9313; PR_15_ challenge: MS023: df = 22, R^2^ = 0.9398; MS049: df = 10, R^2^ = 0.9613; EPZ020411: df = 10, R^2^ = 0.7078; GSK3368715: df = 10, R^2^ = 0.9375). **(G)** Percent metabolic activity after challenging NSC-34 cells with 3 µM of GR_15_ or PR_15_ and dosing with GSK591 (two-way ANOVA with Dunnett’s multiple comparison; n = 6 untreated, n = 3 treated; P values > 0.1657, mean ± s.e.m). The 200 µM dose of GSK591 significantly decreased metabolic activity beyond the effect observed with 3 µM of GR_15_ alone (two-way ANOVA with Sidak’s multiple comparison; n = 6 untreated, n = 3 treated; *P = 0.0261, mean ± s.e.m.) **(H)** Percent metabolic activity after challenging cells with 3 µM of GR_15_ or PR_15_ and dosing with MS094 (two-way ANOVA with Dunnett’s multiple comparison; NS P values >0.5496, mean ± s.e.m.). **(I)** Percent LDH release after challenging cells with 3 µM of GR_15_ or PR_15_ and dosing with MS094 (two-way ANOVA with Dunnett’s multiple comparison; NS P values >0.1650, mean ± s.e.m.). For **(C, D, G, H)**, 100% activity represents untreated NSC-34 cells, and 0% activity represents metabolic activity after 3 µM of GR_15_ or PR_15_ challenge alone. For **(C–F)**, full dose response plots can be found in **Supplementary Figure 1**. For **(A)**, **(C–F)**, a full listing of *n* for each condition can be found in the Supplementary Statistics section of the **Supplementary Material**.

Statistical analyses were performed using Graphpad Prism v.8 and Microsoft Excel. Statistical tests included two-way ANOVAs with Dunnett’s, Sidak’s multiple comparisons tests, one-way ANOVAs with Dunnett’s multiple comparison test, five-parameter logistical regression models to calculate EC50s, and a four-parameter logistical regression model to calculate IC50s. One 1 µM and 0.2 µM data point for MS049 and one 20 µM data point for EPZ020411 in **Figure 1A** were excluded for being outside of two standard deviations of their respective means. A full listing of n values that were not represented in main text figures are presented in **Supplementary Material Tables S2–S6**. Experiments were performed in technical triplicates or quadruplicates, with n values greater than 3 or 4 indicating combination with biological replicates.

## Results

Our lab has reported that exogenous application of GR_15_ and PR_15_ to NSC-34 cells induces cellular toxicity, as measured by WST-1 metabolism, LDH release, BrdU labeling, and Caspase-3 activity (Gill et al., 2019). In the present study, we use the WST-1 metabolism and LDH release assays to evaluate changes in DRP toxicity in the presence of a PRMT inhibitor.

To test the effects of inhibition of various PRMTs in the presence of polyGR or polyPR, we acquired commercially available, small molecule PRMT inhibitors that were capable of inhibiting either ADMe or SDMe. Because multiple PRMTs are capable of catalyzing asymmetric dimethylation of arginine residues, multiple small molecule inhibitors were selected with varying potencies against the various type I PRMTs. These included MS023, GSK715, EPZ020411, and MS049. Because PRMT5 is the more common and abundant of the two Type II PRMTs across and in various cell types (Stopa et al., 2015), we selected a potent and specific inhibitor of PRMT5, GSK591. As a negative control, we used MS094, a previously described structural analog of MS023, which is known to be inert against all PRMT activity (Eram et al., 2016).

We first determined the potency of the PRMT inhibitors in NSC-34 cells using an In-Cell Western (ICW) assay measuring total ADMe for Type I PRMT inhibitors and total SDMe for the Type II PRMT inhibitor (**Table 2** and **Figures 1A, B**). After establishing a working range of concentrations for each inhibitor, we measured metabolic activity (WST1 metabolism endpoint) and cytotoxicity (LDH release endpoint) in NSC-34 cells challenged by various concentrations of GR_15_ or PR_15_ with or without co-incubation with PRMT inhibitors.

**Table 2:** IC50s for inhibition of dimethylation activity and EC50s for abrogation of toxicity caused by GR_15_ or PR_15_ challenge, and chemical structures for each compound tested.

Small molecule	IC50 (µM) ± s.e.	EC50 (µM) ± s.e.				Chemical structure
		3 µM GR15 challenge		3 µM PR15 challenge		
		WST-1	LDH	WST-1	LDH	
MS023	0.545 ± 0.646	0.08 ± 0.02	0.012 ± 0.006	0.132 ± 0.017	~0.029	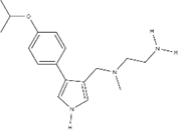
MS049	1.082 ± 0.291	~2.103	~1.765	~1.99	~1.616	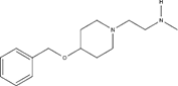
EPZ020411	1.529 ± 0.310	12.01 ± 14.23	1.41 ± 0.118	~2.655	~2.939	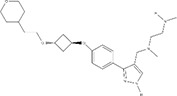
GSK715	0.08 ± 0.441	~0.6724	~0.768	~0.799	~0.269	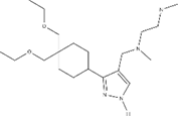
GSK591*	1.914 ± 96.28	–	–	–	–	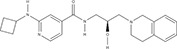
MS094**	–	–	–	–	–	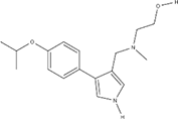
					

IC50s were calculated using a four-parameter logistical regression model, and EC50s were calculated using a five-parameter logistical regression model. Ambiguous values result from curve fits that lack a plateau or constraint.

*Inhibits symmetrical dimethylation.

**Inert analog of MS023.

~indicates value is ambiguous.

We found that the Type I PRMT inhibitors were effective at abrogating the decreased metabolic activity and increased cytotoxicity associated with the application of GR_15_ or PR_15_, with MS023 in particular demonstrating the lowest EC50s (**Table 2** and **Figures 1C–F**). At some concentrations, incubation with Type I PRMT inhibitors resulted in complete rescue of GR_15_ or PR_15_ effects. Most of the inhibitors were inert with regards to LDH and WST1 endpoints in the absence of polyGR and polyPR at concentrations that were effective at abrogating polyGR and polyPR toxicity. The lone exception was EPZ020411. At concentrations at and above 10 µM, MS023, MS049, and EPZ020411 did reduce WST1 metabolism and elevated cytotoxicity, possibly contributing to their bell-shaped dose-response curves (**Supplementary Figures 1A–D** and **Table S1**). MS094, the reported inert analog of MS023, displayed a negligible effect at inhibiting ADMe at 0.1 and 0.2 µM concentrations and did not abrogate the GR_15_- and PR_15_- related toxicity at any concentration (**Figures 1H, I** and **Supplementary Figure 2A**). The Type II PRMT5 inhibitor, GSK591, did inhibit SDMe but did not abrogate the decreased metabolic activity due to GR_15_ and PR_15_ challenge (**Figure 1G** and **Supplementary Figure 3**). These results suggest that the activities of Type I PRMTs contribute to the toxicity produced by GR_15_ and PR_15_.

**Figure 2:**
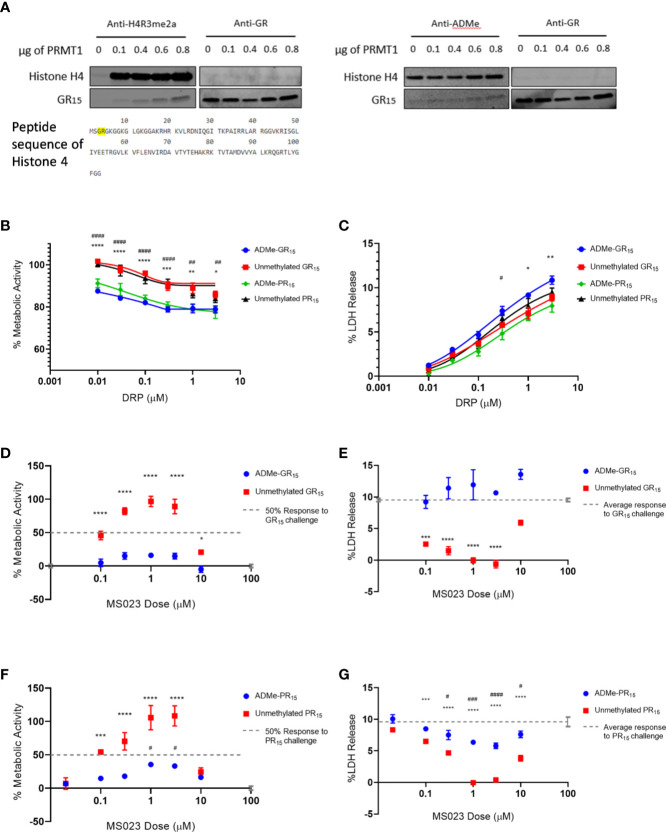
Asymmetrical arginine dimethylation of GR_15_ prevents abrogation of toxicity by MS023. **(A)** Products of in-vitro methylation assay immunoblotted for asymmetrically dimethylated arginine 3 in Histone 4 and GR dipeptide (left) and total asymmetrical arginine dimethylation and GR dipeptide (right). Also shown is the peptide sequence of Histone 4 with the epitope of the H4R3me2a antibody highlighted. Both blots show ADMe of GR_15_ and that it is increasingly dimethylated with increasing amounts of PRMT1. **(B)** Percent metabolic activity after challenging with ADMe-GR_15_ compared to unmethylated GR_15_ (****P < 0.0001, ***P = 0.0002, **P = 0.0013, *P = 0.0281) or ADMe-PR_15_ compared to unmethylated PR_15_ (^####^P < 0.0001, ^##^P = 0.004; two-way ANOVA with Sidak’s multiple comparison; n = 3 for each dose of DRP; mean ± s.e.m.). **(C)** Percent LDH release after challenging with ADMe-GR_15_ compared to unmethylated GR_15_ (**P = 0.0083, *P = 0.0117) or ADMe-PR_15_ compared to PR_15_ (^#^P = 0.0239; two-way ANOVA with Sidak’s multiple comparison; n = 3 for each dose of DRP; NS P > 0.05 mean ± s.e.m.). **(D)** Percent metabolic activity after challenge with 3 µM of ADMe-GR_15_ or unmethylated GR_15_ and dosing with MS023, compared to activity after challenge of 3 µM of GR_15_ alone (two-way ANOVA with Dunnett’s multiple comparison; n = 9 for each dosing group; NS P > 1.638, ****P < 0.0001, *P = 0.0411; mean ± s.e.m.). **(E)** Percent LDH release after challenge with 3 µM of ADMe-GR_15_ or unmethylated GR_15_ and dosing with MS023, compared to release after challenge of 3 µM of GR_15_ alone (two-way ANOVA with Dunnett’s multiple comparison; n = 3 for each dosing group; NS, P > 0.0657, ****P < 0.0001, ***P = 0.0002; mean ± s.e.m.). **(F)** Percent metabolic activity after challenge with 3 µM of ADMe-PR_15_ or unmethylated PR_15_ and dosing with MS023, compared to activity after challenge of 3 µM of PR_15_ alone (two-way ANOVA with Dunnett’s multiple comparison; n = 3 for each dosing group; NS, P > 0.1830, ****P < 0.0001, ***P = 0.0004, ^#^P > 0.0242; mean ± s.e.m.). **(G)** Percent LDH release after challenge with 3 µM of ADMe-PR_15_ or unmethylated PR_15_ and dosing with MS023, compared to release after challenge of 3 µM of PR_15_ alone (two-way ANOVA with Dunnett’s multiple comparison; n = 3 for each dosing group; NS, P > 0.0.2947, ****P < 0.0001, ***P = 0.0007; ^####^P < 0.0001, ^###^P = 0.0004, ^#^P > 0.0273 mean ± s.e.m.). For **(D, F)**, 100% activity represents untreated NSC-34 cells, and 0% activity represents metabolic activity after 3 µM of GR_15_ or PR_15_ challenge alone, respectively. For **(F, G)**, *- PR_15_ + MS023 compared to PR_15_ alone. # - ADMe-PR_15_ + MS023 compared to PR_15_ alone.

One possible interpretation of our data is that the asymmetric dimethylation of the arginine-rich DRPs is essential for arginine-rich DRP toxicity. To evaluate poly-GR as a substrate for Type I PRMT activity we conducted an *in vitro* methylation assay, using recombinant PRMT1 as the enzyme and S-adenosyl methionine (SAM) as the methyl donor group. We used recombinant Histone H4, a known substrate of PRMT1^26^, as a positive control for asymmetrical and symmetrical dimethylation activity. In one experiment, we used antibodies against total ADMe and Histone H4 asymmetrical dimethylation (H4R3me2a) and revealed that GR_15_ is subject to ADMe by PRMT1 in this system and can be increasingly dimethylated when incubated with increasing amounts of PRMT1 (**Figure 2A**). The H4R3me2a antibody was able to detect the ADMe of GR_15_, possibly due to the antibody having been raised against the H4R3me2a epitope containing a methylated arginine 3, which is preceded by a glycine (**Figure 2A**).

After determining that GR_15_ could be arginine methylated, we had ADMe-GR_15_ synthesized. We first compared effects of ADMe-GR_15_ challenge to effects of unmethylated GR_15_ challenge in our LDH and WST-1 assays in NSC-34 cells. ADMe-GR_15_ challenge produced similar levels of cytotoxicity as challenge with unmethylated GR_15_ peptide and caused a significant decrease in cellular metabolic function beyond the effects seen with unmethylated GR_15_ (**Figures 2B, C**). To further elucidate the mechanism behind the protective effects of Type I PRMT inhibitors, we challenged NSC-34 cells with ADMe-GR_15_ and dosed with MS023. In contrast to treatment after challenge with unmethylated GR_15_, MS023 was not able to abrogate the toxicity produced by ADMe-GR_15_ challenge (**Figures 2D, E**). We also had ADMe-PR_15_ synthesized and conducted a similar set of experiments as those with ADMe-GR_15_ and unmethylated GR_15_. Again, we found that ADMe-PR_15_ challenge led to similar levels of cytotoxicity and caused a significantly greater decrease in metabolic activity when compared to unmethylated PR_15_ challenge (**Figures 2B, C**). Interestingly, MS023 co-incubation led to abrogation of toxicity caused by PR_15_ and to, a much lesser extent, toxicity caused by ADMe-PR_15_ (**Figures 2F, G**). Together, these results suggest the importance of asymmetric dimethylation to the toxicity caused by the arginine-rich DRPs however, the post-modification mechanism driving polyGR toxicity could be different than that of polyPR.

## Discussion

The present study reveals that Type I PRMT inhibitors can completely abrogate toxicity produced by exogenous polyGR and polyPR challenge in NSC34 cells and suggests that Type I PRMT inhibition is a potential therapeutic strategy for C9orf72-associated ALS. We also determined that polyGR is subject to ADMe modification, and the ADMe of exogenous polyGR and polyPR is crucial to the toxicity caused by the arginine-rich dipeptide repeats. The partial rescue of ADMe-PR_15_ toxicity by MS023 leaves the question of how the mechanisms of polyGR and polyPR differ. As PRMTs typically react with GAR motifs, methylation and demethylation dynamics could be different between the two DRPs, leading to different responses in the presence of a PRMT inhibitor (Thandapani et al., 2013). Alternatively, it is possible that ADMe-PR_15_ interferes downstream with various PRMT substrates, and the addition of an inhibitor partially prevents those interactions. It is noteworthy that our co-incubation of DRP and Type I PRMT inhibitor produced a bell-shaped dose-response curve, possibly indicating a drastic increase in Type I PRMT activity in hormetic response to the inhibitor (Calabrese et al., 2018). Recent work examining dimethylation found in human cortical tissue suggests symmetric dimethylation of DRPs can extend disease duration (Gittings et al., 2020). As symmetric dimethylation prevents asymmetric dimethylation from occurring on a given substrate, our results are consistent with human pathological findings. Given that our systems were different however, more work in other models needs to be done to better understand how PRMT activity influences C9orf72-mutation mediated neurodegeneration. In summary, our study reveals a novel mechanism that can contribute to arginine-rich DRP toxicity and suggests a possible therapeutic strategy through Type I PRMT inhibition.

## Data Availability Statement

The raw data supporting the conclusions of this article will be made available by the authors, without undue reservation.

## Author Contributions

Conceptualization: AP, AG, and FV. Data curation: AP and AG. Investigation: AP and AG. Validation: AP. Methodology: AP and AG. Project administration: FV. Supervision: FV. Writing (original draft): AP and FV. Writing (review and editing): AG and FV.

## Conflict of Interest

The authors declare that the research was conducted in the absence of any commercial or financial relationships that could be construed as a potential conflict of interest.

## Abbreviations

ALS, amyotrophic lateral sclerosis; FTD, frontotemporal degeneration; DRP, dipeptide repeat protein; RAN, repeatassociated non-AUG; GAR, glycine- and arginine-rich; MMe, monomethylation; ADMe, asymmetric dimethylation; SDMe, symmetric dimethylation; PRMT, protein arginine methyltransferase; SAM, S-adenosyl methionine , ICW, In-Cell Western; IVM, In Vitro Methylation.
