# Supplementary material for: Type I PRMT Inhibition Protects Against C9ORF72 Arginine-Rich Dipeptide Repeat Toxicity
Source: Front Pharmacol. 2020 Sep 8;11:569661. doi: 10.3389/fphar.2020.569661 (PMC7508178; doi:10.3389/fphar.2020.569661)
Supplement: Supplementary file 1 [file DataSheet_1.docx]

**Type I PRMT inhibition protects against C9ORF72 arginine-rich dipeptide repeat toxicity**

Alan S. Premasiri^1*^, Anna L. Gill^1*^, Fernando G.Vieira^1**^

^1^*ALS Therapy Development Institute, 300 Technology Square Suite 400, Cambridge, Massachusetts 02139.*

**These authors contributed equally to this work.*

***Correspondence:* [*fvieira@als.net*](mailto:fvieira@als.net) *Telephone: 617-441-7251 Fax: 617-441-7299*

**Supplementary Material**


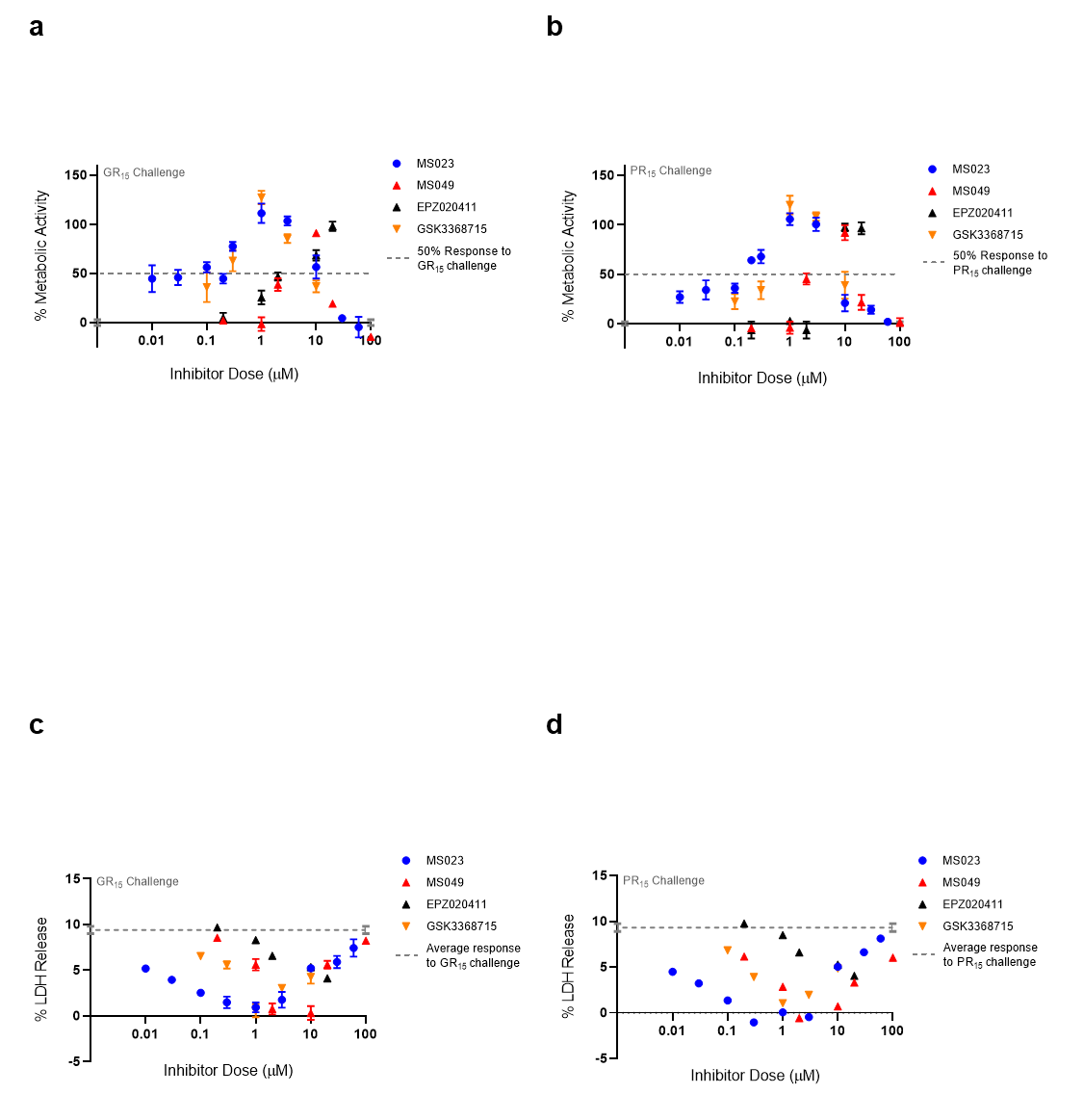

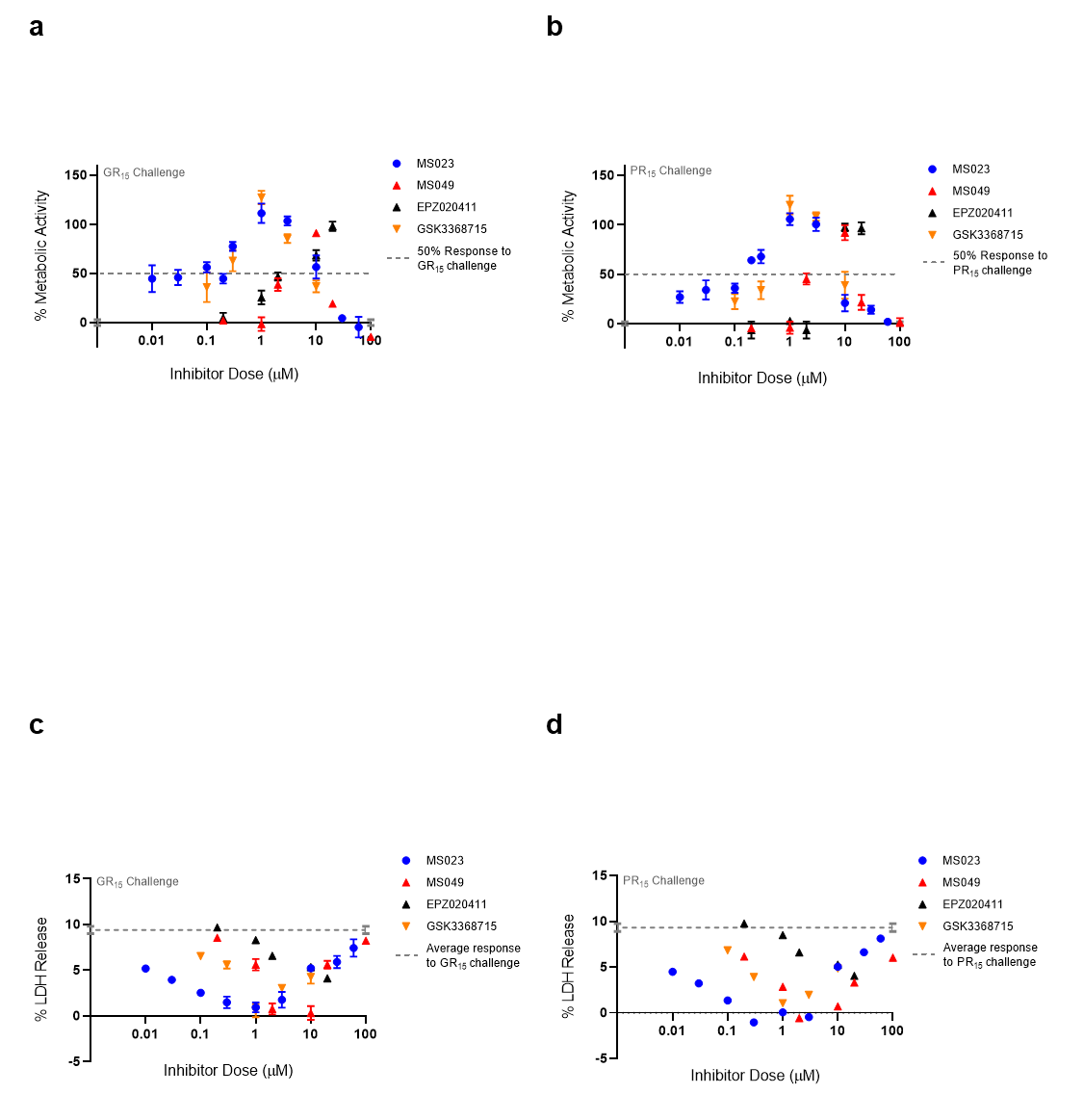


**Supplementary Figure 1**

**Figure S1: Three of four Type I PRMT inhibitor demonstrate a bell-shaped dose-response curve.**

(**a,b**) Full dose-response curves seen in **Fig 1d,e.** of percent metabolic activity after challenging NSC-34 cells with GR^15^ or PR^15^ and dosing with Type I PRMT inhibitors (plotted as mean±s.e.m.). (**c,d**) Full dose-response curves seen in **Fig 1f,g.** of percent LDH release after challenging NSC-34 cells with GR15 or PR15 and dosing with Type I PRMT inhibitors (plotted as mean±s.e.m.). For **a,b,** 100% activity represents untreated NSC-34 cells, and 0% activity represents metabolic activity after 3 µM GR^15^ or PR^15^ challenge alone. A full listing of *n* for each condition can be found in the **Statistics** section of the methods.


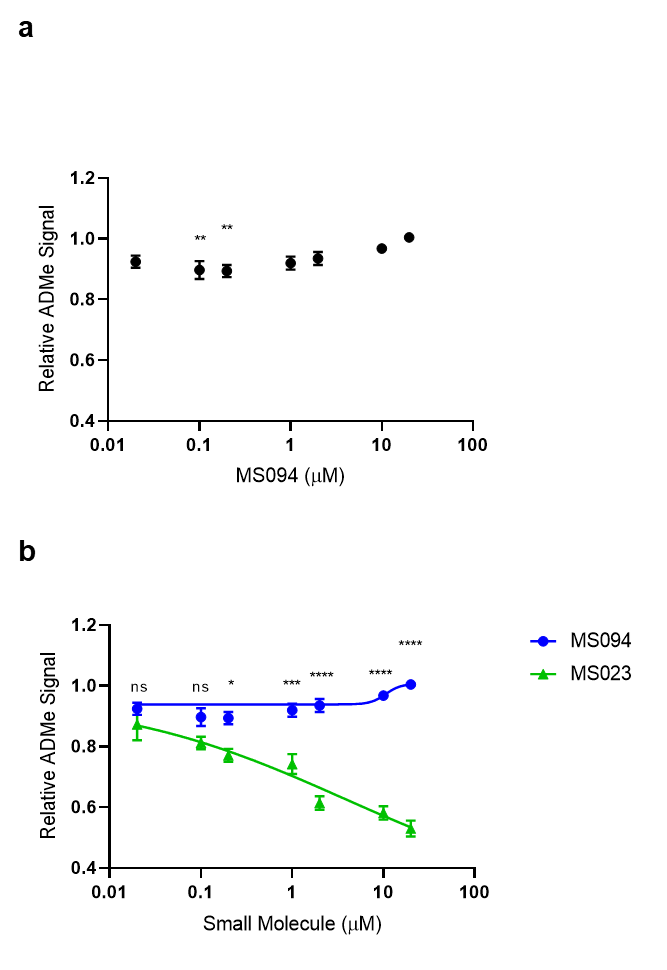


**Supplementary Figure 2**

**Figure S2: MS094 demonstrates minimal to no ability in inhibiting ADMe modification.**

(**a**) Quantified total ADMe signal in NSC-34 cells after dosing with MS094. MS094 caused significant reduction in ADMe signal at 0.1 and 0.2 µM concentrations compared to the signal of the untreated cells (one-way ANOVA with Dunnett’s multiple comparison; n=12 for untreated cells, n=6 for each dosed group; NS P>0.051, control vs 0.1 µM **P=0.0059, control vs 0.2 µM **P=0.0043, mean±s.e.m.). (**b**) Compared total ADMe signal in NSC-34 cells after dosing with MS023 or MS094. MS094 demonstrates a much lower magnitude inhibition of ADMe than that caused by its active analog MS023 (two-way ANOVA with Sidak’s multiple comparison; MS023 data point pulled from **Fig1a.** MS094 data points from (**a**); NS P>0.3656, ****P<0.0001, ***P=0.0003, *P=0.0471, mean±s.e.m.).

**Supplementary Figure 3**

**Figure S3: GSK591 inhibits SDMe modification in NSC-34 cells.**

Quantified total SDMe signal in NSC-34 cells after dosing with GSK591. GSK591 significantly inhibited SDMe modifications at 10 µM and above when compared to the signal of the untreated cells (one-way ANOVA with Dunnett’s multiple comparison; n=10 for untreated cells, n=3 for each dosed group; NS P=0.0703, 10 µM ***P=0.0009, 100 µM ***P=0.0002, **P=0.0029, *P=0.0131, mean±s.e.m.).


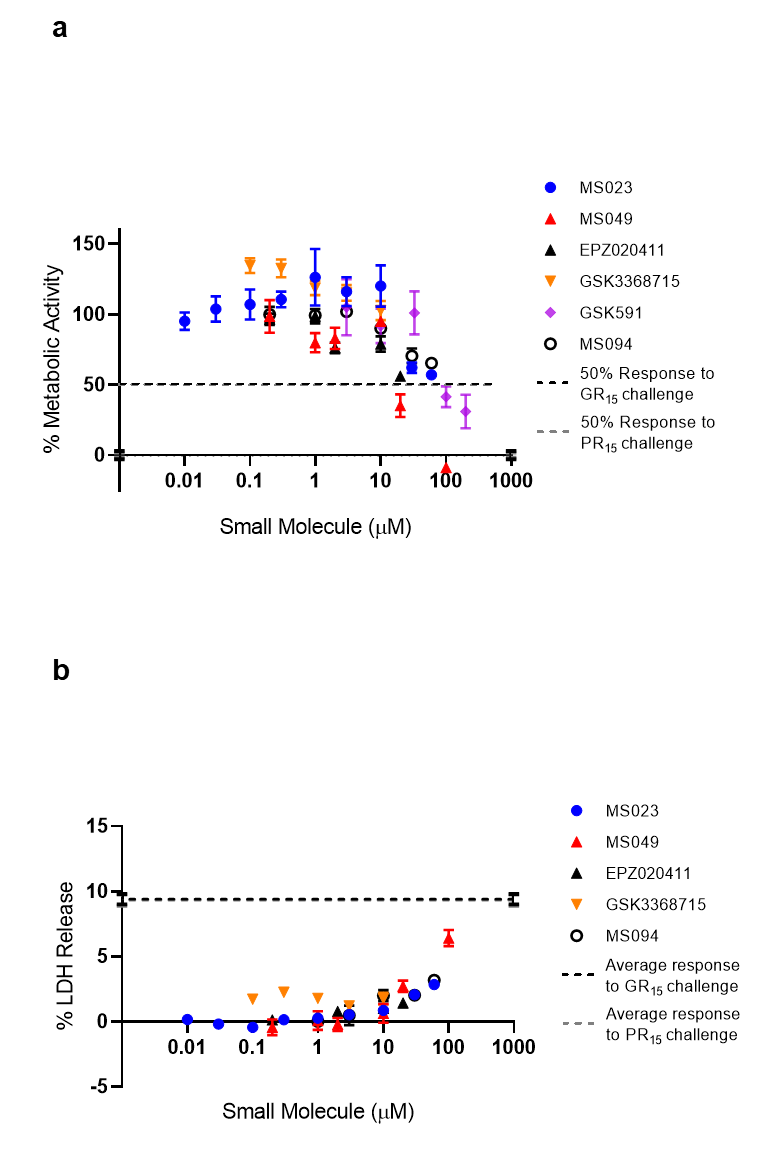


**Supplementary Figure 4**

**Figure S4: Small molecules tested exhibit some toxicity at high doses.**

(**a**) Percent metabolic activity of NSC-34 cells after applying the compounds tested. At concentrations above 10 µM, most compounds go on to show decreased metabolic activity (need table of significances?). (**b**) Percent LDH release by NSC-34 cells after applying the compounds tested. At concentrations above 10 µM, most compounds go on to an increase in LDH release. For **a,** 100% activity represents untreated NSC-34 cells, and 0% activity represents metabolic activity after 3 µM GR15 or PR15 challenge alone. Statistical significance are represented in **Table S1**.


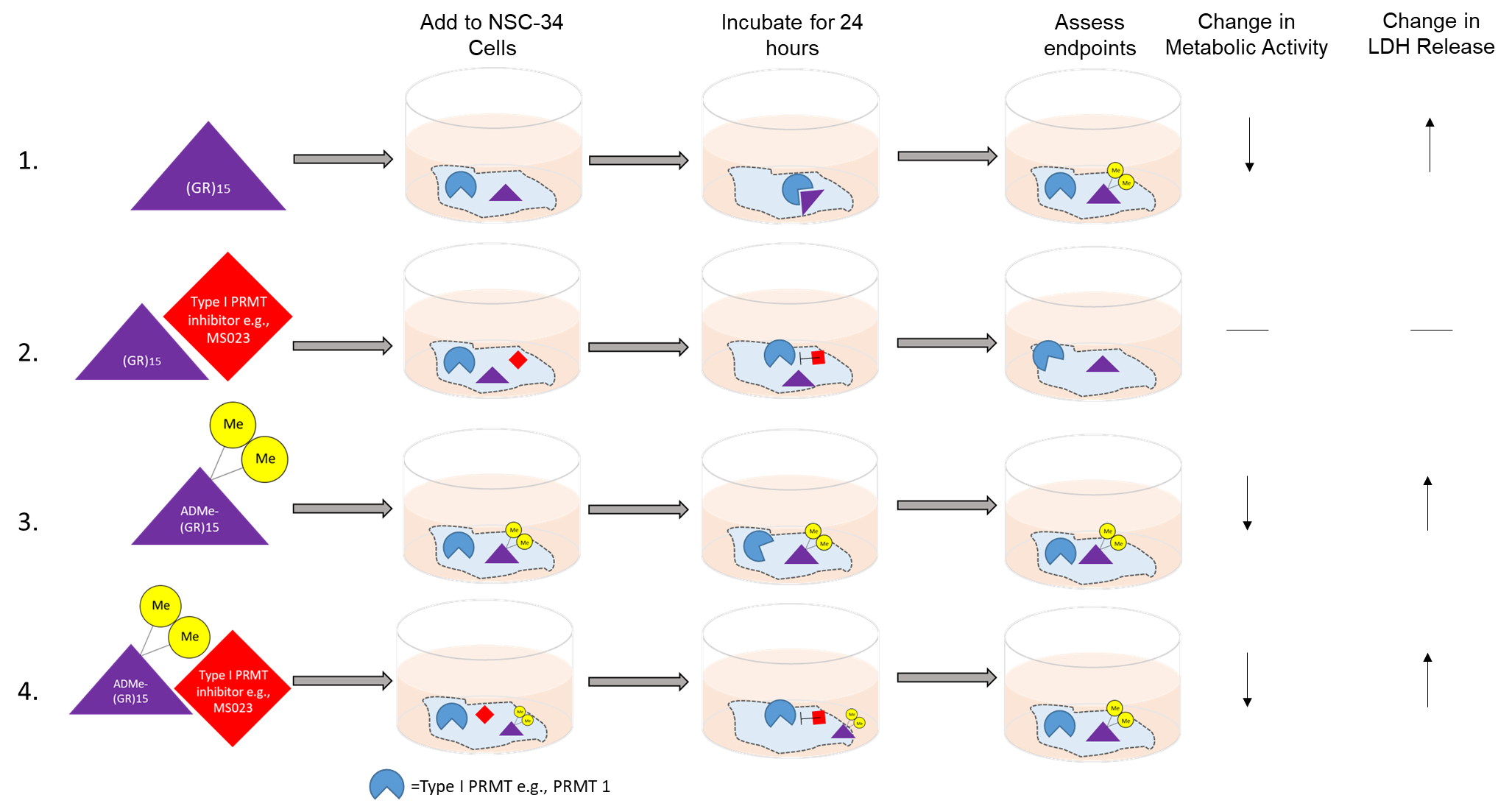


**Supplemental Figure 5**

**Figure S5: Schematic of outcomes of experiments conducted and possible mechanism of toxicity.** Based on the results in the present study the toxicity associated with GR_15_ and PR_15_ (not pictured) could be associated with their ability to be asymmetrically dimethylated after 24 hours of incubation (line 1). When a Type I PRMT inhibitor such as MS023 is added, the toxicity is abrogated, though the exact mechanism by which it happens remains unclear (line 2). When challenging cells with GR_15_ that has been asymmetrically dimethylated, the toxic effects are still present (line 3). However when MS023 was added during the ADMe-GR_15_ challenge, abrogation of toxicity was not observed, and so it is suggested that because GR_15_ was already dimethylated, the PRMT inhibition had no influence on the effects seen (line 4). Taken together, the results suggest that the asymmetric dimethylation of GR_15_ is the driving mechanism of toxicity seen in our assay system.


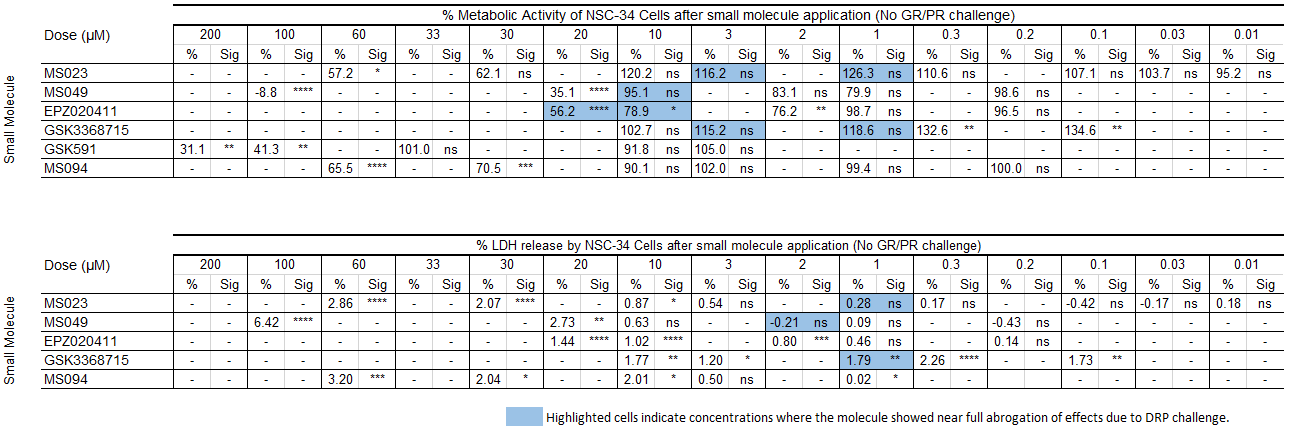


**Table S1. Effects on metabolic activity and LDH release of NSC-34 cells due to molecules in the absence of GR_15_ or PR_15_ challenge.** **** indicates p<0.0001, *** indicates p<0.001, ** indicates p<0.01, * indicates p<0.05. Highlighted cells represent concentrations at which molecules showed near complete abrogation of toxic effects due to GR_15_ or PR_15_ challenge. One-way ANOVAs with Dunnett’s multiple comparisons were used to assess significance. For the WST-1 analysis, percentage of activity was compared to that after DRP challenge. 100% activity represents untreated NSC-34 cells, and 0% activity represents metabolic activity after 3 µM GR_15_ or PR_15_ challenge alone.

**Supplementary Statistics**

**Supplementary Table S2**

| **FIGURE 1A.** | *n* | | | |
| --- | --- | --- | --- | --- |
| Concentration of Inhibitor (µM) | MS023 | MS049 | EPZ020411 | GSK715 |
| 100 | - | 7 | 7 | - |
| 60 | 4 | - | - | - |
| 30 | 4 | - | - | - |
| 20 | 16 | 11 | 11 | 6 |
| 10 | 20 | - | - | 6 |
| 6 | 4 | - | - | - |
| 3 | 4 | - | - | - |
| 2 | 16 | 11 | 11 | 6 |
| 1 | 20 | 11 | 11 | 6 |
| 0.2 | 16 | 11 | 11 | 6 |
| 0.1 | 16 | 4 | 4 | 6 |
| 0.02 | 16 | 4 | 4 | 6 |
| 0 | 42 | 18 | 18 | 12 |

**Supplementary Table S3**

| **FIGURE 1B, S1B** | *n* | | | |
| --- | --- | --- | --- | --- |
| Concentration of Inhibitor (µM) | MS023 | MS049 | EPZ020411 | GSK715 |
| 100 | - | 3 | - | - |
| 60 | 9 | - | - | - |
| 30 | 9 | - | - | - |
| 20 | - | 3 | 3 | - |
| 10 | 12 | 3 | 3 | 6 |
| 3 | 15 | - | - | 6 |
| 2 | - | 3 | 3 | - |
| 1 | 15 | 3 | 3 | 6 |
| 0.3 | 6 | - | - | 6 |
| 0.2 | 3 | 3 | 3 | - |
| 0.1 | 6 | - | - | 6 |
| 0.03 | 3 | - | - | - |
| 0.01 | 3 | - | - | - |
| 0 | 18 | 3 | 3 | 6 |

**Supplementary Table S4**

| **FIGURE 1D, S1B** | *n* | | | |
| --- | --- | --- | --- | --- |
| Concentration of Inhibitor (µM) | MS023 | MS049 | EPZ020411 | GSK715 |
| 100 | - | 3 | - | - |
| 60 | 6 | - | - | - |
| 30 | 6 | - | - | - |
| 20 | - | 3 | 3 | - |
| 10 | 6 | 3 | 3 | 3 |
| 3 | 9 | - | - | 3 |
| 2 | - | 3 | 3 | - |
| 1 | 9 | 3 | 3 | 3 |
| 0.3 | 3 | - | - | 3 |
| 0.2 | 3 | 3 | 3 | - |
| 0.1 | 3 | - | - | 3 |
| 0.03 | 3 | - | - | - |
| 0.01 | 3 | - | - | - |
| 0 | 12 | 3 | 3 | 3 |

**Supplementary Table S5**

| **FIGURE 1E, S1C** | *n* | | | |
| --- | --- | --- | --- | --- |
| Concentration of Inhibitor (µM) | MS023 | MS049 | EPZ020411 | GSK715 |
| 100 | - | 3 | - | - |
| 60 | 6 | - | - | - |
| 30 | 6 | - | - | - |
| 20 | - | 3 | 3 | - |
| 10 | 9 | 3 | 3 | 3 |
| 3 | 9 | - | - | 3 |
| 2 | - | 3 | 3 | - |
| 1 | 9 | 3 | 3 | 3 |
| 0.3 | 3 | - | - | 3 |
| 0.2 | - | 3 | 3 | - |
| 0.1 | 3 | - | - | 3 |
| 0.03 | 3 | - | - | - |
| 0.01 | 3 | - | - | - |
| 0 | 6 | 6 | 6 | 6 |

**Supplementary Table S6**

| **FIGURE 1F, S1D** | *n* | | | |
| --- | --- | --- | --- | --- |
| Concentration of Inhibitor (µM) | MS023 | MS049 | EPZ020411 | GSK715 |
| 100 | - | 3 | - | - |
| 60 | 6 | - | - | - |
| 30 | 6 | - | - | - |
| 20 | - | 3 | 3 | - |
| 10 | 6 | 3 | 3 | 3 |
| 3 | 6 | - | - | 3 |
| 2 | - | 3 | 3 | - |
| 1 | 9 | 3 | 3 | 3 |
| 0.3 | 6 | - | - | 3 |
| 0.2 | - | 3 | 3 | - |
| 0.1 | 3 | - | - | 3 |
| 0.03 | 3 | - | - | - |
| 0.01 | 3 | - | - | - |
| 0 | 6 | 6 | 6 | 6 |

**Tables S2, S3, S4, S5, S6. A full listing of *n* values for figures referenced in the top left corner of each table**. Experiments were performed in technical triplicates or quadruplicates, with n values greater than 3 or 4 indicating combination with biological replicates.
